# “On-Site Family Screening” in Fabry Disease: An Innovative Model for Identifying At-Risk Relatives

**DOI:** 10.3390/jcm15207813

**Published:** 2026-10-10

**Authors:** Erdem Baran, Gizem Kök, Hüseyin Onay

**Affiliations:** 1 Department of Nephrology, Mardin Training and Research Hospital, 47100 Mardin, Türkiye; 2 Department of Nephrology, Yalova Training and Research Hospital, 77200 Yalova, Türkiye; 3 Department of Medical Genetics, Multigen Genetic Diseases Diagnosis Center, 35535 İzmir, Türkiye; gizem.kok.13@gmail.com (G.K.); huseyin.onay@multigen.com.tr (H.O.)

**Keywords:** Fabry disease, family screening, pedigree

## Abstract

**Background:** Fabry disease remains underdiagnosed despite the availability of effective therapies. Although cascade screening is recommended for identifying at-risk relatives, its implementation is often limited by practical and accessibility barriers, resulting in missed or delayed diagnoses. On-Site Family Screening (OSFS) was developed to overcome barriers to conventional cascade screening and improve the identification of previously undiagnosed relatives with Fabry disease. Therefore, this study aimed to implement and evaluate the diagnostic impact of an On-Site Family Screening approach for identifying previously undiagnosed individuals with Fabry disease within an extended family. **Methods:** Following the identification of the index case, at-risk relatives were systematically evaluated through on-site visits at their place of residence. Pedigree analysis was used to identify individuals at risk. Written informed consent was obtained from all participants prior to genetic testing, and molecular analysis of the *GLA* gene was performed. Of all at-risk relatives identified through pedigree analysis, ten at-risk individuals declined participation for personal reasons and were excluded from the analysis. **Results:** A total of 56 individuals were screened. Seventeen previously undiagnosed individuals with Fabry disease were identified, including 10 males and 7 females, corresponding to a diagnostic yield of 30.4%. The mean age at diagnosis was 22.94 years, with an age range from 45 days to 55 years. Notably, two affected male infants aged 45 and 60 days were detected before the development of overt disease manifestations. All affected individuals carried the same pathogenic *GLA* variant, consistent with familial X-linked inheritance. **Conclusions:** In this single extended pedigree, on-site family screening was feasible and facilitated the identification of previously undiagnosed relatives, including individuals at very young and presymptomatic stages. This approach may provide a practical strategy for reaching at-risk individuals who might otherwise remain unscreened. However, as no conventional cascade-screening comparator was included, the incremental benefit of the on-site component cannot be established and requires evaluation in comparative studies.

## 1. Introduction

Fabry disease is an X-linked lysosomal storage disorder caused by pathogenic variants in the *GLA* gene, leading to deficient α-galactosidase A activity and progressive accumulation of glycosphingolipids within lysosomes, affecting multiple organs.

This multisystem disorder affects the renal, cardiovascular, and cerebrovascular systems, as well as the peripheral and central nervous systems, skin, gastrointestinal tract, eyes, and cochleovestibular system, leading to a broad spectrum of clinical manifestations [1,2]. Renal, cardiac, and cerebrovascular involvement are among the major determinants of morbidity and mortality in Fabry disease. However, with advances in the management of renal disease, cardiovascular complications have become the leading cause of death [3,4].

The clinical phenotype and disease severity are influenced by the level of α-galactosidase A activity. The classic form, characterized by less than 1% enzyme activity, typically presents in childhood with early manifestations such as acroparesthesia, angiokeratomas, hypohidrosis, and corneal verticillata, followed by progressive renal, cardiac, and cerebrovascular involvement emerging in early adulthood. In contrast, later-onset forms, associated with residual enzyme activity, present at older ages and are usually characterized by the absence of early manifestations and instead show organ-predominant involvement, most commonly affecting the heart [4,5]. Given the fact that it is an X-linked genetic disorder, hemizygous males tend to be more severely affected than heterozygous females, who show a wide phenotypic spectrum due to X-chromosome inactivation and often with a later onset [6].

Given the highly variable clinical spectrum, severity, and presentation of Fabry disease, the diagnosis is often overlooked or delayed in routine clinical practice. Early manifestations are typically non-specific, including neuropathic pain, gastrointestinal symptoms, hypohidrosis, and fatigue, and may be attributed to more common conditions. The clinical presentation is often fragmented across different specialties, hindering a comprehensive evaluation. Phenotypic variability, particularly in females, further complicates recognition. Moreover, later-onset forms may lack the classic early features and instead present with isolated organ involvement such as renal dysfunction, cardiomyopathy, or cerebrovascular events. These factors frequently result in diagnostic delays exceeding 10 years [7].

Delayed or missed diagnosis in Fabry disease has significant clinical consequences. Progressive organ damage may occur before diagnosis, limiting the effectiveness of therapy once treatment is initiated [8,9]. As reported by Dominique P. Germain et al., enzyme replacement therapy may slow the progression of cardiac and renal disease and improve overall outcomes, with maximal benefit achieved when treatment is initiated early, before the onset of significant organ damage [10]. In addition, delayed diagnosis limits opportunities for informed reproductive planning.

For all these reasons, family-based cascade screening already plays a critical role in Fabry disease. However, in practice, many at-risk relatives do not attend screening, either because they have no symptoms or do not recognize its importance. As a result, diagnosis is frequently missed or delayed.

On-site family screening aims to address an important gap in current screening strategies by directly reaching at-risk individuals who are unlikely to present to healthcare institutions for evaluation. In this way, it may increase participation in screening programs and contribute to earlier recognition of the disease.

In this study, a pedigree-based on-site family screening model was implemented to improve the detection of previously undiagnosed Fabry disease cases by directly reaching at-risk family members.

## 2. Methods

This study was designed as a retrospective, pedigree-based familial cascade screening and descriptive cohort study in Mardin City, Southeastern Türkiye. The index case was a 45-year-old woman with no documented family history of Fabry disease. She first presented with moderate kidney dysfunction, characterized by a serum creatinine level of 1.2 mg/dL and an estimated glomerular filtration rate (eGFR) of 70 mL/min/1.73 m^2^, along with marked proteinuria measuring 750 mg/day. Clinical assessment identified typical neuropathic manifestations, including acroparesthesia, as well as mild sensorineural hearing impairment. Cardiac examination showed no signs of left ventricular hypertrophy and brain MRI findings were negative for cerebrovascular abnormalities.

The definitive diagnosis of Fabry disease was established following deficient enzyme activity testing and subsequent comprehensive *GLA* sequencing, including analysis of all coding exons and exon–intron boundaries, which confirmed the pathogenic c.335G>A (p.R112H) variant. Intravenous enzyme replacement therapy (ERT) with agalsidase beta was promptly initiated at the standard therapeutic dose of 1 mg/kg every two weeks.

Before initiating OSFS, the index patient and her family were informed about the proposed family screening approach, and their willingness to facilitate the screening of relatives was assessed. Written informed consent was obtained from the index patient before initiating contact with at-risk relatives and sharing information for the purpose of family screening. Following identification of the index case, potentially at-risk relatives were identified through pedigree analysis, and the pedigree was subsequently reviewed by the medical geneticists involved in the study to determine eligibility for screening.

Importantly, the medical team did not independently initiate contact with at-risk relatives. The index patient and her family first informed potentially eligible relatives about the possibility of family screening and facilitated initial communication with those who expressed an interest in participating. Relatives who were willing to undergo screening subsequently contacted, or agreed to be contacted by, the clinician, and the OSFS process was initiated on the basis of their voluntary willingness to participate. Thereafter, Dr. Erdem Baran and the screening team contacted these individuals directly to coordinate clinical assessment, genetic counseling, and sample collection. Participation in clinical assessment and genetic testing was entirely voluntary. Ten individuals identified as being at risk through pedigree analysis declined participation for personal reasons. Consequently, these individuals did not undergo clinical assessment or genetic testing and were excluded from the final analysis.

After verbal agreement to participate, participants’ addresses were obtained and home visits were scheduled. A laboratory nurse visited eight separate households over a two-day period. In some households, several family members were present together by prior arrangement. During the home visits, participants were informed about the screening process, and written informed consent was obtained before sample collection. Peripheral blood samples were collected into EDTA tubes by the laboratory nurse, appropriately labeled, and transported to the laboratory for molecular genetic analysis.

Participants were informed that their test results would subsequently be communicated through the clinician. Following completion of genetic testing, the reports were provided to the clinician, and individual genetic counseling sessions were arranged with a medical geneticist involved in the study to discuss the results, associated risks, and recommendations for further evaluation and follow-up.

Genomic DNA was extracted from peripheral blood leukocytes using conventional methods. According to the laboratory testing algorithm, comprehensive *GLA* sequencing, including all coding exons and exon–intron boundaries, was performed for all participants. The coding regions and exon-intron boundaries of the *GLA* gene were amplified through polymerase chain reaction. Library preparation was performed using the Nextera XT sample preparation kit, and sequencing was conducted on the Illumina MiniSeq platform (Illumina, San Diego, CA, USA). Sequencing data were visualized using the Integrative Genomics Viewer (IGV), and variant interpretation was carried out through multiple databases, including HGMD, ClinVar, and GnomAD. Variants were evaluated and classified in accordance with the 2015 guidelines of the American College of Medical Genetics and Genomics (ACMG).

### Statistical Analysis

Descriptive statistics were used to summarize demographic and clinical characteristics. Continuous variables are presented as mean ± standard deviation or median (interquartile range), whereas categorical variables are presented as frequencies and percentages. Statistical analyses were performed using IBM SPSS Statistics for Windows, version 31.0 (IBM Corp., Armonk, NY, USA).

## 3. Results

Of the at-risk relatives identified through pedigree analysis, 10 individuals declined participation and did not undergo clinical assessment or genetic testing. A total of 56 individuals from a single family were screened with the “On-Site Family Screen” approach. Seventeen previously undiagnosed cases of Fabry disease were identified, including ten males and seven females (Table 1, Figure 1 and Figure 2). In this single extended family pedigree, Fabry disease was detected in 17 previously undiagnosed individuals out of 56 relatives screened using the OSFS method, corresponding to a diagnostic yield of 30.4%. The mean age of the affected individuals was 22.94 years, ranging from 45 days to 55 years. Affected individuals were detected across multiple generations, including those with mild or previously unrecognized clinical features. Several of these individuals had not undergone prior evaluation despite being at risk (Table 1).

The flow diagram illustrates the sequential OSFS process, including the identification of at-risk relatives through pedigree analysis, contact and assessment for participation, informed consent, genetic testing, and final diagnostic classification. Ten at-risk relatives declined participation and did not undergo genetic testing. Among the 56 individuals who participated in the screening and underwent molecular genetic testing, 17 were diagnosed with Fabry disease and 39 tested negative for the familial pathogenic *GLA* variant.

Clinical manifestations varied considerably among the 17 newly diagnosed relatives (Table 2). Most affected males were identified at an early or presymptomatic stage and showed no overt renal, cardiac, or neurological manifestations at the time of screening. Among female relatives, clinical expression was more heterogeneous, ranging from an asymptomatic phenotype to mild or non-specific manifestations, including fatigue, occasional neuropathic pain, dizziness, and proteinuria. Genetic analysis confirmed that all 17 affected relatives carried the familial pathogenic c.335G>A (p.R112H) GLA variant, despite the observed variability in clinical expression. α-Galactosidase A activity and lyso-Gb3 levels were available for affected individuals. In relatives for whom these biochemical measurements were not performed, the diagnosis was established by molecular genetic testing demonstrating the familial pathogenic c.335G>A (p.R112H) GLA variant. Following diagnosis, affected individuals underwent comprehensive multi-organ evaluation and follow-up tailored to their age and clinical findings. In particular, the two affected male infants, aged 45 and 60 days, were referred to a pediatric metabolic disease center for long-term surveillance and parental counseling regarding the timing of treatment initiation (Table 2).

## 4. Discussion

In this single extended pedigree, OSFS identified 17 previously undiagnosed individuals with Fabry disease among 56 screened relatives, corresponding to a diagnostic yield of 30.4%. This proportion should be interpreted within the context of the structure and X-linked inheritance pattern of this particular pedigree and should not be considered an expected diagnostic yield of OSFS in general. Nevertheless, the identification of previously unrecognized affected relatives across successive generations illustrates the potential utility of proactive family-based screening for identifying individuals who had not previously undergone specialized evaluation.

Studies conducted in Türkiye on Fabry disease family screening have reported high diagnostic yields originating from a single index case through cascade screening. To the best of our knowledge, this study represents one of the largest single-pedigree family screening experiences reported from Türkiye. In a family screening study by Daniş et al., 13 affected individuals were identified through cascade screening initiated from an index patient [11]. Likewise, Kisa et al., in a pediatric cohort, reported the identification of up to 15 affected relatives from a single index case [12]. Similar observations have been reported in family screening cohorts in Russia; these studies also identified a large number of new patients through family screening initiated from index cases [13]. However, direct comparison with these previous studies is limited by differences in pedigree structure and screening strategies. These findings further highlight the critical importance of systematic family-based screening approaches in hereditary lysosomal storage disorders, particularly in populations where delayed diagnosis and intrafamilial under-recognition remain substantial challenges.

One potential advantage of the OSFS model is that it may help address practical barriers that limit traditional cascade screening. Geographic distance, limited access to specialized centers, socioeconomic difficulties, insufficient disease awareness, and reluctance to undergo evaluation frequently reduce participation among at-risk relatives. Germain et al. highlighted that cultural, educational, and infrastructural constraints continue to represent significant challenges for family-based genetic testing programs globally [14]. By directly visiting family members within their own living environment, the OSFS approach may help reduce some of these barriers and facilitate the evaluation of individuals who might otherwise not present for screening.

Another important finding of our study was the marked phenotypic heterogeneity among affected relatives despite the presence of the same familial pathogenic GLA variant. Most affected males were identified at early or presymptomatic stages without overt organ involvement, whereas female relatives exhibited a broader clinical spectrum, ranging from an asymptomatic phenotype to mild neurological manifestations and proteinuria. Such variability is consistent with the influence of residual enzyme activity and X-chromosome inactivation on disease expression, particularly in females, in whom α-galactosidase A activity may be normal or near normal, underscoring the importance of molecular genetic testing for diagnosis [15]. This intrafamilial heterogeneity is also supported by Rusu et al., who described seven relatives carrying the same pathogenic GLA variant but exhibiting renal involvement ranging from preserved kidney function and hyperfiltration to proteinuria and advanced chronic kidney disease [16]. Collectively, these findings demonstrate that molecular confirmation establishes genetic status but does not reliably predict the extent or severity of organ involvement. Therefore, individualized renal, cardiac, and neurological assessment and longitudinal surveillance remain essential for affected relatives identified through family screening.

The mean age at diagnosis in our cohort was 22.94 years, and several affected relatives were identified at young or presymptomatic stages, including two male infants. Although this mean age was lower than that reported in some historical Fabry disease cohorts, this difference cannot be interpreted as a quantitative reduction in diagnostic delay because the present cohort was identified through an established pedigree and included infants and asymptomatic relatives. Rather, these findings suggest that proactive family screening may facilitate the identification of affected individuals before overt disease manifestations develop.

Early diagnosis has critical therapeutic implications in Fabry disease. Progressive glycosphingolipid accumulation eventually leads to irreversible renal, cardiac, and cerebrovascular injury, significantly reducing quality of life and life expectancy. Multiple studies have demonstrated that early initiation of enzyme replacement therapy or chaperone therapy may slow disease progression and improve long-term outcomes, whereas delayed treatment is associated with diminished therapeutic benefit [17]. Beyond facilitating early diagnosis and longitudinal surveillance, molecular family screening may also inform genotype-directed therapeutic decisions. In a recent real-world cohort of 87 adults with Fabry disease, McCarron et al. reported that migalastat was generally well tolerated and associated with stable renal and cardiac parameters and sustained reductions in plasma lyso-Gb3 among patients with amenable *GLA* variants [18]. These findings suggest that relatives identified through OSFS may be considered for pharmacological chaperone therapy when the familial variant is confirmed to be amenable; nevertheless, treatment eligibility and therapeutic decisions should be determined individually based on variant amenability and the patient’s clinical profile.

The identification of two affected male infants aged 45 and 60 days raises important ethical and clinical considerations that merit specific discussion. Current international guidelines do not recommend initiating enzyme replacement therapy in presymptomatic male infants, as the long-term benefit of treatment prior to the onset of organ involvement has not been established. However, early identification confers several advantages: it enables prospective monitoring for emerging organ damage, allows for timely parental and family counseling, and facilitates informed reproductive decision-making for at-risk relatives [19]. In our cohort, both infants were referred to a pediatric metabolic disease center, where a structured surveillance protocol was initiated in accordance with current recommendations. From an ethical standpoint, the disclosure of genetic diagnoses in minors requires careful consideration of the child’s future autonomy and the psychological impact on the family. In this context, pre-test and post-test genetic counseling was provided to the parents of both infants, addressing the implications of the diagnosis, the natural history of Fabry disease in hemizygous males, and the anticipated timeline for therapeutic decision-making. These cases further illustrate the importance of integrating pediatric metabolic expertise and psychological support into the OSFS workflow when young children are identified among at-risk relatives.

Despite its advantages, OSFS is a labor-intensive and time-consuming process requiring travel organization, pedigree reconstruction, genetic counseling, and coordination among multiple family members. Ethical considerations, including privacy concerns and communication within families, may also complicate implementation. Nevertheless, the identification of previously undiagnosed affected individuals in this pedigree suggests that OSFS may provide a useful approach to family screening despite these logistical challenges [20].

This study has several limitations. It represents the experience of a single pedigree and a single-center screening initiative, limiting generalizability. In addition, a number of at-risk relatives declined participation, which may have led to an underestimation of the true prevalence of Fabry disease within the family. Furthermore, the absence of a comparator group undergoing conventional cascade screening precludes assessment of the incremental benefit attributable specifically to the on-site component. No formal health-economic assessment of the OSFS approach was performed. Finally, long-term clinical outcomes of individuals identified through OSFS were not available at the time of analysis.

## 5. Conclusions

In conclusion, in this single extended pedigree, OSFS was feasible and facilitated the identification of 17 previously unrecognized individuals with Fabry disease, including individuals identified at young and presymptomatic stages. By enabling direct access to at-risk relatives, this approach may provide a practical strategy for family-based screening. However, because no conventional cascade screening comparator was included, the incremental benefit attributable specifically to the on-site component cannot be established. Future multicenter comparative studies are needed to evaluate the reproducibility, clinical utility, and cost-effectiveness of OSFS in different healthcare settings.

## Figures and Tables

**Figure 1:**
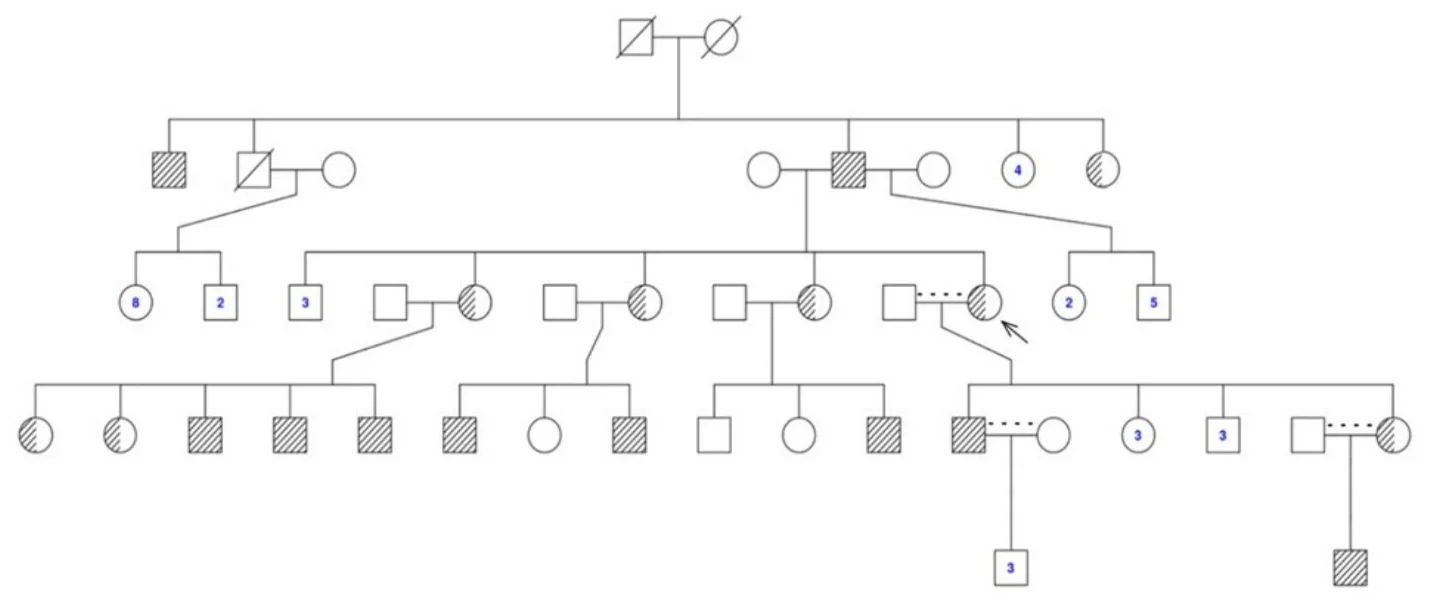
Pedigree of the family after performing OSFS. Squares represent males and circles represent females. Fully shaded squares indicate hemizygous males carrying the pathogenic *GLA* variant, whereas half-shaded circles indicate heterozygous females carrying the variant. Numbers within symbols denote multiple individuals with the same phenotype/genotype represented collectively. The arrow indicates the proband. Slashed symbols indicate deceased individuals.

**Figure 2:**
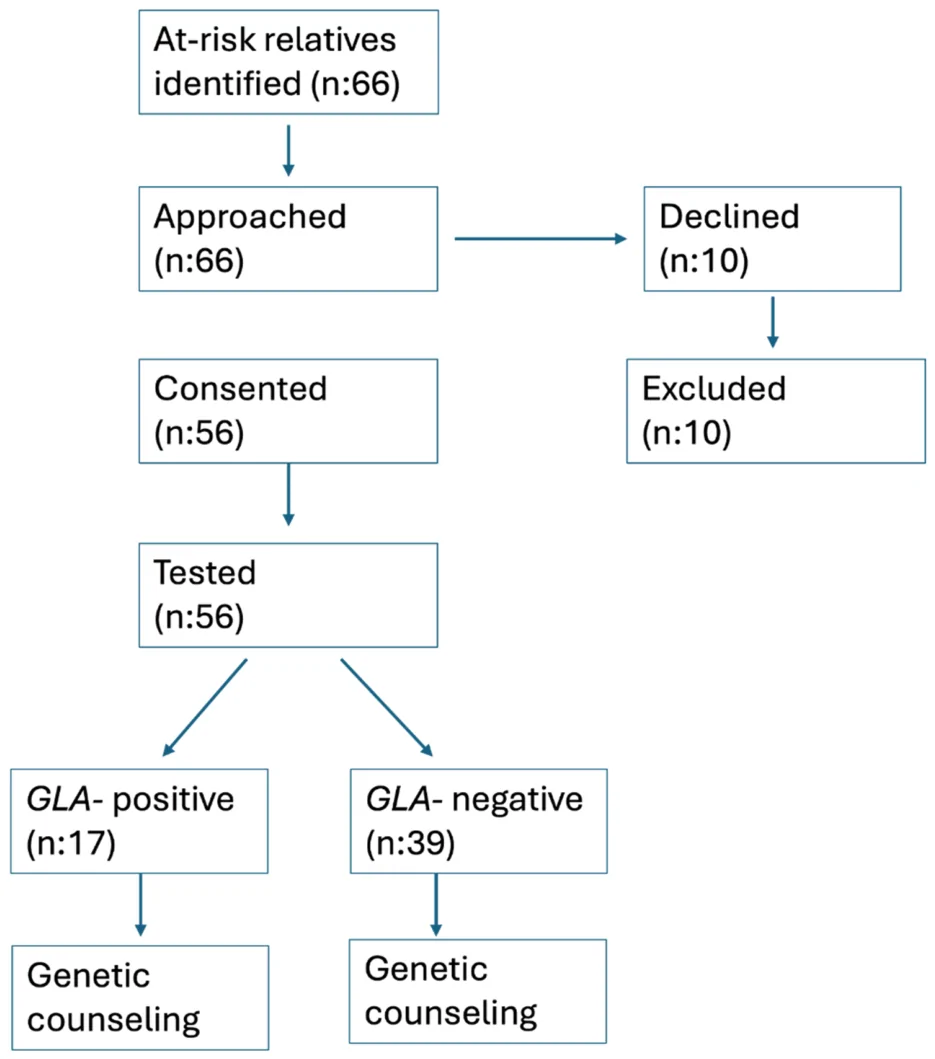
Participant Flow Diagram of the On-Site Family Screening (OSFS) Process for Fabry Disease.

**Table 1:** Diagnostic Performance and Demographic Characteristics of the On-Site Family Screening (OSFS) Cohort.

Variable	Value
Individuals screened	56
At-risk individuals refusing participation	10
Newly diagnosed cases	17
Diagnostic yield	30.4%
Male	10
Female	7
Mean age	22.94 years
Age range	45 days–55 years
Affected infants	2

**Table 2:** Individual Demographic, Biochemical, and Clinical Characteristics of Relatives Diagnosed With Fabry Disease Through OSFS.

Patient	Age at Diagnosis	Sex	α-Galactosidase A Activity(µmol/L/h)	Lyso-Gb3 (ng/mL)	Renal Findings	Cardiac Findings	Neurological/Other Manifestations	Clinical Status/Management
1	45 days	Male	0.1	5.5	None identified	None identified	None identified	Pediatric metabolic follow-up
2	60 days	Male	0.1	—	None identified	None identified	None identified	Pediatric metabolic follow-up
3	3 years old	Female	1	3	None identified	None identified	None identified	Observation without treatment
4	5 years old	Male	0.1	8.7	None identified	None identified	None identified	Observation without treatment
5	7 years old	Male	0.87	2	None identified	None identified	None identified	Observation without treatment
6	10 years old	Female	0.1	3.6	None identified	None identified	None identified	Observation without treatment
7	10 years old	Male	0.1	2.3	None identified	None identified	None identified	Observation without treatment
8	10 years old	Male	0.1	1.6	None identified	None identified	None identified	Observation without treatment
9	18 years old	Male	0.1	1.6	None identified	None identified	None identified	Observation without treatment
10	21 years old	Female	—	—	None identified	None identified	Fatigue	Observation without treatment
11	30 years old	Male	—	—	None identified	None identified	Presymptomatic	Observation without treatment
12	37 years old	Female	—	—	None identified	None identified	Fatigue	Observation without treatment
13	38 years old	Female	—	—	None identified	None identified	Occasional neuropathic pain	Observation without treatment
14	38 years old	Male	—	—	None identified	None identified	Presymptomatic	Observation without treatment
15	44 years old	Female	2.8	—	Proteinuria	None identified	Dizziness	Observation without treatment
16	46 years old	Female	—	—	Proteinuria	None identified	Occasional neuropathic pain	Enzyme replacement therapy
17	55 years old	Male	—	—	None identified	None identified	Presymptomatic	Observation without treatment

Note: All affected relatives carried the familial c.335G>A (p.R112H) GLA variant. Dashes indicate individual-level data not available in the current manuscript dataset.

## Data Availability

The genetic variant data generated during this study are individual, identifiable clinical results produced by an accredited third-party diagnostic laboratory as part of routine patient care. Due to patient privacy and the small, identifiable nature of the extended pedigree described here, these data are not suitable for public deposition and are not publicly available. De-identified variant-level information may be made available by the corresponding author upon reasonable request, subject to institutional and laboratory data-sharing policies and after approval by the responsible ethics committee.

## Abbreviations

ACMG	American College of Medical Genetics and Genomics
eGFR	Estimated glomerular filtration rate
ERT	Enzyme replacement therapy
*GLA*	Alpha-galactosidase A (gene)
HGMD	Human Gene Mutation Database
IGV	Integrative Genomics Viewer
MRI	Magnetic resonance imaging
NGS	Next-generation sequencing
OSFS	On-Site Family Screening
PCR	Polymerase chain reaction
